# Surgical Management of Penile and Preputial Neoplasms in Equine with Special Reference to Partial Phallectomy

**DOI:** 10.1155/2013/891413

**Published:** 2013-09-08

**Authors:** Awad Rizk, Esam Mosbah, Gamal Karrouf, Mohamed Abou Alsoud

**Affiliations:** ^1^Surgery, Anaesthesiology and Radiology Department, Faculty of Veterinary Medicine, Mansoura University, Mansoura, 35516 Dakahlia, Egypt; ^2^King Fahd Medical Research Center, King Abdulaziz University, 21589 Jeddah, Saudi Arabia; ^3^Biology Science Department, King Abdulaziz University, 21589 Jeddah, Saudi Arabia

## Abstract

Penile and preputial neoplasia in horses occurs infrequently and represents diagnostic and therapeutic challenges. The present study was carried out on a total number of 21 equids (14 stallions and 7 donkeys) suffered from different penile and preputial neoplasia. Diagnosis of neoplasms was based up on history of the case, clinical examination as well as histopathological evaluation. Animals with penile and preputial neoplasms were underwent local excision and partial phallectomy with a slightly modified version of the techniques described by William's. The diagnosed neoplasms were penile and preputial squamous cell carcinomas (SCCs; *n* = 15); sarcoid (*n* = 4); a-fibrosarcoma; and a melanoma. Local excision was curative in all cases except 5 stallions with SCCs. These stallions had extensive damage of the glans penis, free part of the penis and the inner lamina of the internal fold of the prepuce, and they underwent a partial phallectomy with successful outcome. Follow-up information was obtained by visit and telephone inquiries. In conclusion, penile and preputial neoplasms are commonly encountered in elderly male horses and SCCs are the most common type affecting male external genitalia. Partial phallectomy is effective for management of equine neoplasia if they are confined to the glans and body of the penis and there is no proximal spread or involvement to regional lymph nodes.

## 1. Introduction

Penile and preputial neoplasia is more prevalent in equine than in other domestic animals; they occupied about 6 and 10% of all neoplasms in equine [1, 2]. Squamous cell carcinomas (SCCs) are the most common form of penile and preputial neoplasia [3–5]; however sarcoids, squamous cell papillomas, melanomas, fibromas, fibrosarcoma, and haemangiomas have been reported [3, 4, 6, 7]. Penile and/or preputial neoplasia can cause discomfort and in advanced cases can result in systemic disease [8–10].

SCCs are the commonest neoplasm involving the penis, prepuce, or both [6]. It represents 20% of all equine tumours, making it the second most common tumour in horses. It is a malignant tumour mainly affecting adult horses [5, 6, 11, 12]. SCCs of the penis and prepuce are locally invasive but usually show only low-grade malignancy [2]. These tumours may metastasize to regional lymph nodes if not treated aggressively. Recurrence after surgical excision can be expected in 17–25% of cases [2, 4, 5]. Long-standing SCCs can interfere with coitus or normal protrusion and retraction of the penis and most penile SCCs involve the glans penis (53–80%) [4, 11, 12].

Fibrosarcoma is a malignant tumor derived from fibrous connective tissue and characterized by immature proliferating fibroblasts or undifferentiated anaplastic spindle cells. It is unusual mesenchymal tumor in male genitalia of man [13], horse [4], and cattle [14].

Equine sarcoid is a locally aggressive, fibroblastic skin tumour, which can appear as a flat or cauliflower-like growth either singularly or multiple growths, usually in young horses [15]. Sarcoids have been clinically categorized into several groups including verrucous, fibroblastic, mixed verrucous and fibroblastic, occult, nodular, and malevolent. Some of these subtypes can occur with any frequency on the equine penis and prepuce [15–17].

Melanocytic tumours have been reported to affect all parts of the penis and prepuce other than the glans [4, 9, 16]. Malignant melanomas were seen predominantly in older nongrey horses and melanocytic nevi occurred in young horses of all coat colours [7, 16].

Recently, a standard protocol has been proposed to aid treatment selection of horses with penile and preputial tumours. Treatments were ranged from minimally invasive interventions (e.g., topical use of 5-fluorouracil) to radical surgery [2]. Surgical modalities include local excision, segmental posthioplasty, partial phallectomy, partial phallectomy and sheath ablation, and en bloc penile and preputial resection with penile retroversion [2, 18–21].

Local excision is indicated to remove small, solitary tumours, such as focal SCCs, squamous papilloma, and sarcoid, where there is no evidence of metastasis to regional lymph nodes or invasion to deeper structures. Local excision is usually curative for horses with melanocytic nevi and solitary dermal melanomas [22, 23].

Partial phallectomy, such as Scott's, Williams', or Vinsot's techniques, is indicated only if the distal portion of the penis (the glans and shaft of the penis) is involved and there is no proximal spread and without involvement of the prepuce and regional lymph nodes [4, 5]. Involvement of prepuce, urethra, and cavernous tissue are indications for performing more radical surgery [21].

The present study aimed to examine various types of penile and preputial neoplasia in horses and donkeys in our region and investigate the results of the surgical intervention using a modified version of Williams's technique for partial phallectomy. 

## 2. Materials and Methods

The present study was carried out on a total number of 21 equids (14 horses and 7 donkeys) and diagnosed different penile and preputial neoplasia (Tables 1 and 2). These animals were admitted to Mansoura Veterinary Teaching Hospital of the Faculty of Veterinary Medicine, Mansoura University, Mansoura, Egypt. The age was ranged from 4 to 12 years old for horses and 2 to 10 years old for donkeys (Table 2). Diagnosis was based on history of the case and clinical examination of each mass including location, size, age as well as histopathological examinations.

### 2.1. Clinical Examination

Clinical examination was performed of the penis and prepuce. Examination of the penis itself and the internal fold of the prepuce required sedation with intravenous detomidine 1% (Domosedan, Pfizer) in a dose of 0.01 mg/kg. body weight. Superficial inguinal lymph nodes were examined.

### 2.2. Surgical Intervention

Each animal was held off feed for 12 hrs preoperatively and received procaine penicillin (22,000 IU/kg, IM) and phenylbutazone (2.2 mg/kg, IV; Phenyloject, Adwia, Egypt) prior to general anesthesia. 

Anesthesia was induced and maintained by infusion of a freshly prepared mixture of 3 mg medetomidine (3 *μ*g/mL), 40 mg midazolam (0.04 mg/mL) and 2 gm ketamine (2 mg/mL) dissolved in 1 liter of dextrose 5% in animals either underwent local excision or partial phallectomy.

### 2.3. Local Excision

 The observed lesions were surgically resected through a wide surgical excision in the surrounding healthy tissues by 1-2 cm. Two Penrose drains were placed, exiting the incision laterally. Primary closure of the incision was accomplished using a series of interrupted vertical mattress sutures using prolene (Braun-Aesculap, Tuttlingen, Germany) no. 0 and appositional sutures (simple interrupted).

### 2.4. Partial Phallectomy with a Modified Williams's [24] Technique

This modified technique was performed in five stallions diagnosed SCCs at the glans penis (3 horses), free part of the penis, and the inner lamina of the internal fold of the prepuce (2 horses).

Horses were positioned in dorsal recumbency. A catheter was placed in the urethra and advanced into the bladder as a guide to aid in dissection of the urethra and to evacuate urine at the end of the procedure. Soft-roll gauze was used to tie the free part of the penis to an overhead support to facilitate aseptic preparation. The glans and proximal free part of the penis were covered in a sterile bandage. A latex tourniquet was placed proximal to preputial ring. The penis, prepuce, and the surrounding ventral abdomen were aseptically prepared and the area was draped. 

**Figure 1:**
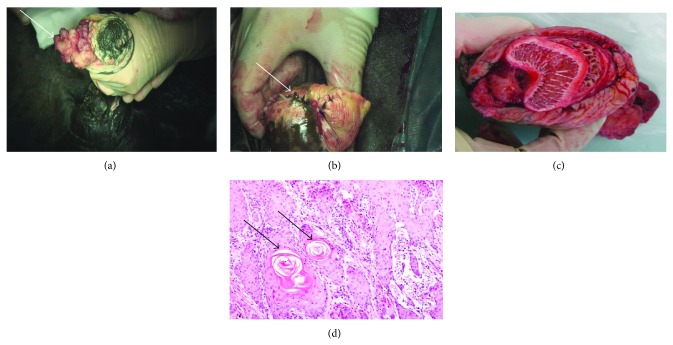
Cauliflower-like SCC at the glans penis of horse (white arrow). The incised edges of the urethral mucosa were opposed to the epithelial edges of the sides of the triangle with simple continuous suture (white arrow; b). Stroma of partial phallectomy (c). Histological structure of SCCs showing small aggregates, irregular islands, and nests of neoplastic keratinocytes (black arrow) that proliferate downward from the epidermis and invade the subepithelial stroma of the dermis (H & E ×400).

A triangular incision (base 3 cms and sides 5 cms) was made through the penile skin on the ventral midline of the penis, with the apex pointing in a proximal direction. The base of the triangle was proximal to corona glandis and it was the site of penile transection. This triangle was bluntly dissected. A longitudinal incision was made into the urethra from the apex to the base of the triangle. The incised edges of the urethral mucosa were opposed to the epithelial edges of the sides of the triangle with simple continuous sutures (Figure 1(b)) using polydiaxanone no. 0 (PDS, Braun-Aesculap, Tuttlingen, Germany). The base of the triangle was left unsutured. The urethral catheter was removed, and the penis was obliquely transected at the base of the triangle in a craniodorsal direction (Figure 1(c)). The tunica albuginea of the dorsum of the corpus cavernosum penis was sutured to the tunical albuginea of the urethral groove with equidistant simple interrupted sutures using PDS no 0. The urethral mucosa was sutured to the penile epithelium with simple interrupted sutures using the same suture material. Open covered castration was performed in these 5 stallions at the same time of surgery.

### 2.5. Postoperative Care

The preoperative antibiotic and anti-inflammatory were continued for 5 days. A prophylactic dose of antitetanic serum was injected. Wound dressing using povidone iodine was performed twice daily. Animals were confined to a stall without exercise for 5 days after surgery.

### 2.6. Histopathological Examination

Samples of the excised masses were fixed in neutral buffered formalin 10%. The specimens were sectioned at 5 microns, stained with hematoxylene and eosine according to Bancroft et al. [25].

### 2.7. Long-Term Followup

Follow-up information was obtained by visit and telephone inquiries. Owners were questioned as to the horse's general health status, recurrence of masses, complications from the surgical procedure, and the ability of the operated animals to urinate freely.

## 3. Results

In the present study, 21 equids (14 horses and 7 donkeys) showed various types of penile and preputial neoplasms (Table 1). The diagnosed neoplasms were penile (*n* = 9) and preputial (*n* = 6) SCCs, penile fibrosarcoma (*n* = 1), penile (*n* = 1) and preputial (*n* = 3) sarcoid, and preputial melanoma (*n* = 1) which was detected in a stallion with SCCs (the 2 neoplasms in the same animal) who underwent partial phallectomy (Table 2). 

SCCs were detected in 15 equids representing 71.4% of the total recorded cases (Tables 1 and 2). The animals suffered nonhealing erosions and granulation tissue of the penile and preputial surfaces. The tumour appeared as a proliferative cauliflower-like or solid mass (Figure 1(a)). Microscopically SCCs consisted of small aggregates, irregular islands, and nests of neoplastic keratinocytes that proliferate downward from the epidermis and invade the subepithelial stroma of the dermis. Frequent findings include keratin formation, horn pearls, mitoses, and cellular atypia (Figure 1(d)).

Local excision was curative in 10 cases with SCCs. The other five stallions showed 5-7 mm pedunculated masses present on glans penis, 4 mm friable mass within the urethral fossa, and 5 mm mass located on the free part of the penis. Preputial bleeding and an intense, pungent odour were found. These 5 stallions underwent partial phallectomy with A modified Williams technique. Good recovery without recurrence was obtained in three horses one year after surgery, while the other 2 horses showed regrowth of the neoplasia at the stump of the penis 5 and 7 months after surgery. Surgical excision was performed in the two cases for a second time with successful recovery without recurrence one year after surgery.

Two horses and one donkey had been treated by local excision of the SCCs and showed regrowth of the neoplasia 3 months after surgery. Surgical excision was performed in the two cases for a second time with successful recovery without recurrence 9 months and 16 months after surgery.

**Figure 2:**
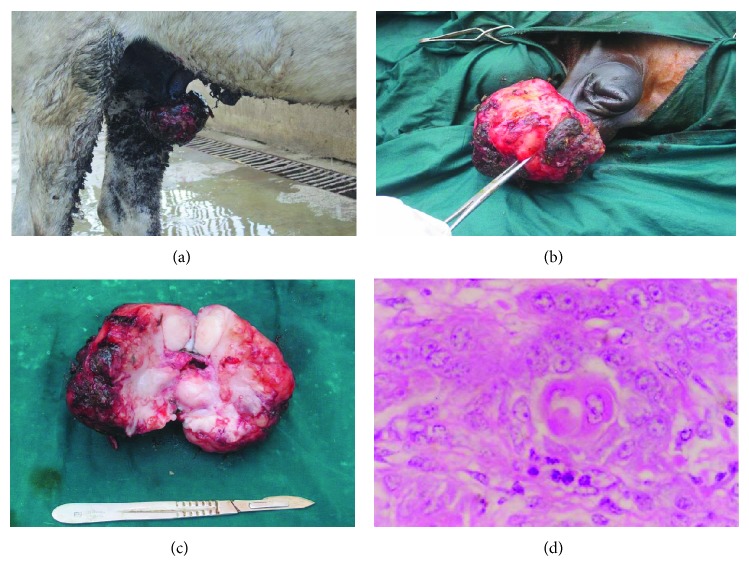
Fibroblastic sarcoid on the prepuce of a donkey (a & b). The mass after surgical excision (c). Micrograph for sarcoids in donkeys showing hyperplasia of the epithelium, elongation of rete pegs, ulceration of the epithelium, inflammatory cell infiltration, and thrombosis and fibromatous growth in the dermis (d) H & E., original magnification ×400.

Penile fibrosarcoma was diagnosed in a stallion representing 4.7% of the total neoplastic cases. Examination of the penis revealed a large ulcerated soft tissue mass on the shaft of the penis. Microscopically, tumor cells showed mesenchymal origin characterized by spindle cells in nodular whorls or streams and bundles, high nuclear to cytoplasm ratio, and oddly shaped large nuclei, without inflammatory component and necrosis. Successful outcome was obtained after local excision without recurrence 13 months after surgery. Penile and preputial sarcoids were detected in 2 donkeys and 2 horses with an average age (2–5 years) and representing 19.2% of the total neoplastic cases. They appeared singular or multiple cauliflower like growths (Figures 2(a), 2(b) and 2(c)). Microscopically, there was a classical streaming and interlacing spindle cell population, and “picketfence” appearance, at the epithelial interface, and long, thin, dissecting rete ridges are typical characteristic features to equine sarcoids (Figure 2(d)). Three operated animals showed no recurrence of the masses after local excision while one donkey showed recurrence 3 months after surgery which appeared smaller in size than the former one. Surgical excision was performed for a second time with successful recovery without recurrence 9 months after surgery. 

**Figure 3:**
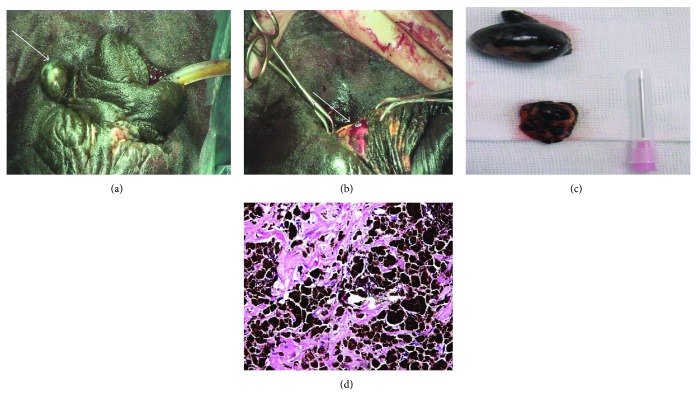
Solitary, firm, nodular, and hairless melanotic melanoma (white arrow) at the prepuce of horse (a). The mass was surgically excised (white arrow; b). The mass after surgical excision (c). Melanotic melanoma showing intracytoplasmic dense brownish black pigment (melanin) which obscures the cellular details; besides, fibrous tissue trabeculae were seen among them (d) H & E, original magnification ×520.

A case of melanoma was detected in one stallion with SCC (the 2 neoplasms were in the same animal) who underwent partial amputation of the penis. It appeared as a solitary, firm, nodular, and hairless mass with intact skin at the prepuce (Figure 3(a)). The mass was surgically excised with successful outcome without recurrence one year after surgery (Figures 3(b) and 3(c)). Microscopically, the tumor characterized by a variety of cell patterns ranging from sheets to streams and nests of melanocytes shifts the diagnosis to a melanotic melanoma. Cell morphology varies from being epithelioid to spindle-shaped. Melanin pigmentation was mild and it was seen interspersed between the tumour cells (Figure 3(d)).

Postoperative oedema was observed in 3 animals operated via partial phallectomy. It receded after 4 days and urination was not impeded. Intermittent haemorrhage associated with urination was observed frequently 2 weeks after partial phallectomy in one stallion, but it stopped spontaneously without the need for further surgery or any other specific therapy.

## 4. Discussion

Partial phallectomy is generally regarded as a salvage procedure in stallions for purposes other than breeding [26]. Various techniques of phallectomy have been described [24, 27–29]. With all of these cases a Williams' technique was employed by creating a triangular rather than linear incision, and, positioning the base of the triangle distally, the risk of urethral stricture is reduced [24, 30]. The technique of partial phallectomy which is performed in this study was similar to that described by Williams [24], but, instead of apposing the incised edges of the urethral mucosa to the epithelial edges of the sides of the triangle with simple interrupted sutures, simple continuous sutures were performed which incorporate and compress the cavernous tissue of the corpus cavernosum penis. In addition, instead of transection of the penis in a craino-distal direction, the penis was obliquely transected at the base of the triangle in a craniodorsal direction, so that the dorsum of the penile stump was longer than the ventrum.

Surgical intervention aims to remove lesions and adequate margins to reduce the risk of recurrence [2]. Partial phallectomy with or without retroversion has been described in the standing horse [29, 31] and avoids the costs and risks involved with general anesthesia [31]. It was not deemed suitable for partial phallectomies in this study, due to the size and temperament of the horses, and the increased risk to the surgeon and assistant.

Unsuccessful outcome (i.e., recurrence or incomplete removal) of SCCs treated by partial phallectomy is not uncommon (43.5%). This was possibly as a result of the limitation to the amount of tissue that can be removed combined with the frequent presence of the more proximal part of the penis or the prepuce of premalignant tumors, such as papillomas that may subsequently transform to SCCs [5]. The annulus of the inner preputial fold is generally given as the anatomical border up to which phallectomy can be performed because of the risk of suture failure if more extensive tissue removal is attempted. However, in some cases the primary tumor may be too close to this anatomical landmark to allow excision of an adequate margin [4, 5].

Two horses and one donkey treated via local excision of the SCCs underwent a second surgery three months after the first surgery due to the regrowth of the mass. This was consistent with the finding of [4, 5]. Tumour recurrence is possible when the surgeon fails to remove all neoplastic cells from the surgical site; therefore, wide surgical excision is recommended. Primary closure is indicated, if possible. The prognosis for complete recovery after local excision is excellent if adequate tissue margins are obtained [30].

Castration of stallions several weeks before phallectomy is advisable to help in modification of behavior [4, 16, 30, 32, 33]. In this study, animals that had been treated with partial amputation of penis underwent open covered castration at the same time of surgery. Penile and preputial neoplasia in horses occur infrequently, but they represent diagnostic and therapeutic challenges because lesions can be present for a considerable period before clinical signs are noted by owners [11]. The clinical signs of various types of penile tumours are not specific and can result from the primary tumor or secondary inflammatory processes. Symptoms commonly include irregularities of the penile surface, sanguineous or purulent discharge followed by disturbed micturition and edema of the prepuce. Other signs include skin excoriation, frequent protrusion of the penis, and lameness assuming a wide-based stance [4, 25, 34, 35].

SCCs appear as single or multiple, raised and ulcerated. Advanced lesions often appear as florid, cauliflower-like masses with areas of necrosis, ulceration, and hemorrhage. They are the most common form of penile and preputial neoplasms in horses [36]. Recurrence of SCCs in the present study after surgical excision is 20%. Long-standing SCCs can interfere with coitus or normal protrusion and retraction of the penis, and most penile SCCs involve the glans penis (53–80%) [4, 11, 12]. Horses that present with penile or preputial SCCs tend to be older, pony breeds [11, 12, 35].

In this study, three horses that operated via a slightly modified phallectomy were cured without incidence of recurrence one year after surgery. While two cases showed a local recurrence of the masses at the stump of the penis and tunica albugenia, this may be due to that those horses were affected by a more advanced stage of disease at the time of surgery than that was appreciated. This might have involved spread to inguinal lymph nodes or the presence of tumour in the penile or preputial tissues remaining after surgery. In none of these horses, the superficial lymph nodes were palpably enlarged at the time of surgery, suggesting that palpation of the lymph nodes may not be effective in identifying early metastasis [4, 5, 12]. These results were in agreement with that reported by [4, 5, 11, 12]. 

Haemorrhage is frequently observed after phallectomy especially during and immediately after urination [12, 37]. The prevention of haemorrhage is important to reduce postoperative swelling and the risk of wound dehiscence. In this study, haemostasis was achieved by placing simple continuous sutures through the tunica albuginea of the dorsum of the corpus cavernosum penis and the tunica albuginea of the urethral groove, for compressing the erectile bodies. The use of an elastrator and specialised latex tubing at the site of proposed amputation was described [29], and it was left in situ postoperatively, to prevent haemorrhage and produce necrosis of the distal penis. This is likely to be an extremely painful procedure. Although haemorrhage may increase the risk of dehiscence, it is usually of minor significance [12, 37, 38]. 

In this study, local excision of the smaller penile and preputial neoplasms which are not invading the tunica albugenia was considered the radical method of treatment. It is economic, fast, and mostly curative. However, this sort of treatment has a high risk of incomplete removal followed by recurrence [4]. If the operation is performed earlier with a wide safety margin, treatment may be satisfactory with good prognosis; however, when the action happens later, the prognosis becomes poor due to large capacity of metastatic tumor and recurrence [4, 5].

In this study, a case of fibrosarcoma was recorded on the body of the penis which is an uncommon tumor of the external genitalia of male horses [4]. Van Den Top et al. [4] recorded such neoplasm on the external fold of the prepuce of a horse.

Four animals in this study showed penile and preputial sarcoids. In the surgically treated cases, 75% of the treated sarcoids (3 cases) did not recur throughout the time of followup. This was considered a satisfactory success rate. However, this success rate could probably be due to the seat of the sarcoid on prepuce and removal of a wide margin of normal tissues around the sarcoid to avoid the recurrence of the lesion. This was in agreement with previous results obtained by [15, 39–41].

Melanoma is a common, generally slow growing, locally invasive tumour, estimated to occur in approximately 80% of ageing grey horses [9, 23]. Garvican et al. [9] reported a series of cases of penile or preputial melanomas resulting in systemic metastasis. There were metastases of other equine penile neoplasia [12]. Our case appeared as solitary, firm, nodular, and hairless mass with intact skin at the prepuce. It was surgically excised with successful outcome. Follow-up report indicated a good recovery and the horse was in a good health condition.

## 5. Conclusion

- In conclusion, the results of this study confirmed that partial phallectomy with a slightly modified version of Williams's technique is an effective therapy for horses if neoplasia is confined to the glans and body of the penis and there is no proximal spread without involvement of the prepuce and regional lymph nodes.
- Penile and preputial tumours are commonly encountered in elderly male horses except sarcoids in 2–5 years old, and SCCs are by far the most common type of tumour affecting male genitalia.
- Fibrosarcoma is uncommon tumours of the equine penis.

## Figures and Tables

**Table 1:** Showing type, breed, number, and percentage of penile (*n* = 11) and preputial (*n* = 10) neoplasms in stallion (*n* = 14) and Jack donkey (*n* = 7).

Types	Penis		Prepuce		Total	%
	Stallion	Jack	Stallion	Jack		
SCCs	6	3	4	2	15	71.4
Fibrosarcoma	1	—	—	—	1	4.7
Sarcoid	1	—	1	2	4	19.2
Melanoma	—	—	1	—	1	4.7

Total number	8	3	6	4	21	100

**Table 2:** Showing a patient data including animal species, age, tumour types, treatment, recurrence, followup, second treatment, second recurrence, and followup of penile (*n* = 11) and preputial (*n* = 10) neoplasms in stallion (*n* = 14) and Jack donkey (*n* = 7).

Subject	Animal type	Age (year)	Penile or preputial	Tumor type	Treatment	Recurrence	Followup	Second treatment	Recurrence	Followup
1	H	6	Pre	Me	Loc	No	12 Mo	—	—	—
2	D	4	Pe	SCC	Loc	No	12 Mo	—	—	—
3	H	6	Pe	SCC	Wil	Yes	5 Mo	Loc	No	12 Mo
4	D	8	Pe	SCC	Loc	No	12 Mo	—	—	—
5	H	10	Pe	SCC	Wil	Yes	7 Mo	Loc	No	12 Mo
6	D	5	pr	Sarcoid	Loc	No	8 Mo	—	—	—
7	H	11	Pr	SCC	Wil	No	12 Mo	—	—	—
8	H	11	Pr	SCC	Loc	No	12 Mo	—	—	—
9	H	9	Pe	Fib	Loc	No	13 Mo	—	—	—
10	D	10	Pe	SCC	Loc	No	9 Mo	—	—	—
11	H	11	Pr	SCC	Loc	No	10 Mo	—	—	—
12	D	2	Pr	Sarcoid	Loc	Yes	3 Mo	Loc	No	9 Mo
13	H	12	Pr	SCC	Loc	Yes	3 Mo	Loc	No	16 Mo
14	H	8	Pr	SCC	Wil	No	14 Mo	—	—	—
15	H	9	Pe	SCC	Loc	No	16 Mo	—	—	—
16	H	12	Pe	SCC	Wil	No	8 Mo	—	—	—
17	D	7	Pe	SCC	Loc	No	11 Mo	—	—	—
18	H	11	Pe	SCC	Loc	Yes	3 Mo	Loc	No	9 Mo
19	H	4	Pe	Sarcoid	Loc	No	6 Mo	—	—	—
20	D	8	Pe	SCC	Loc	No	9 Mo	—	—	—
21	H	3	Pr	Sarcoid	Loc	No	12 Mo	—	—	—

H: horse, D: donkey, Loc: local excision, Wil: Williams, Pe: penile, Pr: preputial, Fib: fibrosarcoma, Me: melanoma, Mo: Month.
